# Inner workings of thrombolites: spatial gradients of metabolic activity as revealed by metatranscriptome profiling

**DOI:** 10.1038/srep12601

**Published:** 2015-07-27

**Authors:** J. M. Mobberley, C. L. M. Khodadad, P. T. Visscher, R. P. Reid, P. Hagan, J. S. Foster

**Affiliations:** 1 Department of Microbiology and Cell Science, University of Florida, Space Life Science Lab-Exploration Park, Merritt Island, FL 32953; 2 Department of Marine Sciences, University of Connecticut, Groton, CT 06340; 3 Rosenstiel School of Marine Sciences, University of Miami, Miami, FL, 33149.

## Abstract

Microbialites are sedimentary deposits formed by the metabolic interactions of microbes and their environment. These lithifying microbial communities represent one of the oldest ecosystems on Earth, yet the molecular mechanisms underlying the function of these communities are poorly understood. In this study, we used comparative metagenomic and metatranscriptomic analyses to characterize the spatial organization of the thrombolites of Highborne Cay, The Bahamas, an actively forming microbialite system. At midday, there were differences in gene expression throughout the spatial profile of the thrombolitic mat with a high abundance of transcripts encoding genes required for photosynthesis, nitrogen fixation and exopolymeric substance production in the upper three mm of the mat. Transcripts associated with denitrification and sulfate reduction were in low abundance throughout the depth profile, suggesting these metabolisms were less active during midday. Comparative metagenomics of the Bahamian thrombolites with other known microbialite ecosystems from across the globe revealed that, despite many shared core pathways, the thrombolites represented genetically distinct communities. This study represents the first time the metatranscriptome of living microbialite has been characterized and offers a new molecular perspective on those microbial metabolisms, and their underlying genetic pathways, that influence the mechanisms of carbonate precipitation in lithifying microbial mat ecosystems.

Microbialites are one of the oldest known ecosystems on Earth, dominating the planet for more than 80% of its history^1^, yet little is known about the molecular mechanisms underlying these lithifying communities. Much like their ancient counterparts, modern microbialites are globally distributed, and actively grow in a wide range of freshwater, marine and hypersaline environments^2^,^3^,^4^,^5^,^6^,^7^,^8^. Microbialites are formed through the coordinated activities of microbes, resulting in the trapping and binding of sediment grains, as well as biologically induced precipitation of calcium carbonate^9^,^10^,^11^. These ancient ecosystems are differentiated by their underlying carbonate microstructure properties with two dominant types. Iterative laminated deposits are classified as stromatolites, whereas unlaminated, clotted structures are referred to as thrombolites^12^,^13^. The exact microbial mechanisms and processes that lead to these disparate microbialite fabrics are not yet known.

Until recently, the study of microbialite formation had been the purview of geologists and biogeochemists, with much of the research focusing on stromatolitic microbialites^9^,^14^,^15^,^16^,^17^,^18^,^19^,^20^,^21^,^22^,^23^,^24^,^25^,^26^,^27^. These previous studies have shown that through their metabolisms and cell-to-cell interactions the associated microbial mat community, creates both spatial and temporal biogeochemical gradients that influence calcium carbonate precipitation and dissolution within microbialite systems^10^,^11^,^28^. Based on this prior work, several microbial functional guilds have been delineated that are thought to influence carbonate mineralization in microbialites and include: oxygenic and anoxygenic phototrophs, aerobic heterotrophs, sulfate reducers, sulfide oxidizers, and fermenters^18^,^19^,^29^,^30^,^31^.

Together, these guilds are thought to impact the net precipitation potential of calcium carbonate by influencing both the saturation index and cycling of exopolymeric substances (EPS)^11^. The saturation index is determined by the availability of free calcium and carbonate ions, the latter of which is in part governed by the pH. Carbonate availability is influenced by the net microbial metabolic processes that either increase the pH (e.g., photosynthesis; some types of sulfate reduction) to promote precipitation or decrease the pH (e.g., aerobic respiration; sulfide oxidation)^28^,^32^. The EPS matrix represents an extension of the cell and may be an important means by which microbialite mat communities “engineer” their proximate physicochemical environment. EPS production within microbialite communities is also critical for carbonate precipitation, as this material can strongly bind to cations^33^ and serve as a carbon source for heterotrophic metabolism^18^,^34^, provide structural stability to the community in physically dynamic systems^35^, and function as nucleation sites for calcium carbonate precipitation^36^,^37^,^38^,^39^.

These previous geologic and biogeochemical studies on modern microbialites are now being complemented with molecular-based approaches. Surveys of amplicon libraries targeted to the 16S and 18S rRNA genes within lithifying microbial mat systems have identified a taxonomically diverse consortium, with all the previously identified functional guilds represented^3^,^4^,^5^,^24^,^26^,^31^,^40^,^41^,^42^,^43^,^44^,^45^,^46^,^47^,^48^,^49^. More recently, a few studies have examined the metagenome of actively growing microbialites^4^,^6^,^50^,^51^,^52^,^53^. In these studies, the taxa and potential biochemical pathways associated with photosynthesis, sulfate reduction, sulfide oxidation, heterotrophy and fermentation have been delineated, as are pathways associated with carbohydrate metabolism, specifically the production and degradation of EPS^4^,^52^. However, the spatial organization and expression of the functional genes associated with these biologically induced mineralization pathways remain unknown.

In the present study, molecular and microelectrode profiling were used to generate a spatial portrait of the taxonomic and metabolic activity within a living microbialite at midday, coinciding with the peak of photosynthesis. The marine thrombolites of Highborne Cay, The Bahamas (Fig. 1a,b) were targeted for this study, as the processes associated with the formation of unlaminated lithifying communities are not well understood and this ecosystem represents one of the few ecological sites where both thrombolites and stromatolites are juxtaposed within a few meters. Together, this study represents the first metatranscriptomic survey of a lithifying microbial ecosystem, thereby enabling an important first look into molecular networking that occurs within these complex communities. Additionally, metatranscriptomic analyses will help delineate the specific roles that each taxa and functional group plays in coordinating the spatial gradients of metabolic activity that ultimately lead to biologically induced organomineralization.

## Results and Discussion

**Figure 1:**
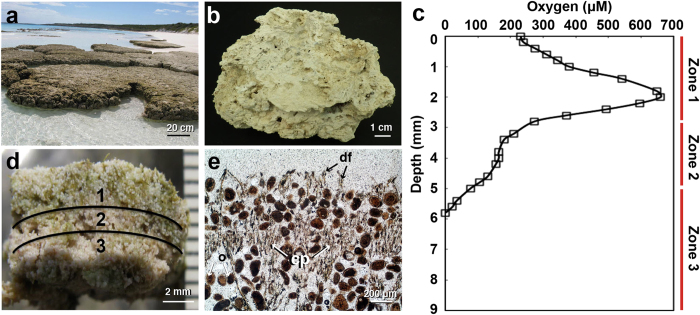
The thrombolites from Highborne Cay, The Bahamas. (**a**) Thrombolite platforms located in the intertidal zone. Bar = 20 cm. (**b**) Cross-section of thrombolite build-up depicting unlaminated microstructure. Bar = 1 cm. (**c**) Microelectrode profiling of oxygen concentration throughout the thrombolitic mat. (**d**) Cross-section of the thrombolitic mat delineating the three zones examined in this study. Bar = 2 mm. (**e**) Petrographic thin section of the upper 2 mm of the thrombolitic mat revealing numerous *Dichothrix* sp. filaments (df). Attached to the filaments are deposits of carbonate precipitate (cp) interspersed within the oolitic sand grains (o). Bar = 200 μm. Photos were taken by authors of this study.

Metatranscriptomic libraries were generated for three zones within the vertical profile of thrombolitic mats collected from Highborne Cay, The Bahamas at midday (12 pm) (Fig. 1c,d). The spatial zones were delineated by vertical microelectrode profiling of oxygen concentration between 12 pm and 1 pm. Three replicate profiles showed a similar distribution of oxygen, with a single peak (625–700 μM) occurring ca. 2 mm beneath the surface of the mat (Fig. 1c). Zone 1, the supersaturated oxic zone, included the upper 3 mm of the thrombolitic mat and was marked by a sharp increase and rapid decrease of oxygen, suggestive of high rates of oxygen production and consumption. Zone 1 was also marked by a high relative abundance of calcified cyanobacterial filaments, previously identified through morphology and 16S rRNA gene sequencing as *Dichothrix* spp.^23^,^47^. Bundles of these vertically oriented filaments were enriched in the supersaturated oxic Zone 1 and petrographic thin sections showed deposition of calcium carbonate along the *Dichothrix* sheaths (Fig. 1e). Zone 2 was a transitional zone between 3–5 mm beneath the surface (Fig. 1d) where oxygen concentrations decreased from 300 μM to 100 μM. This slower rate of decrease with depth suggested a less active aerobic community, which is typical for the heterogeneous distribution of the microbial activities in clotted thrombolites^24^. The lower Zone 3 ranged from 5–9 mm and was predominantly anoxic with only oxygen present in the 0–6 mm depth horizon. Beneath 9 mm the thrombolite transitioned to hard, carbonate substrate.

Based on the microelectrode profiling, two metatranscriptomic libraries were created for each zone including one containing total RNA extracts from the mat and the other consisting of enriched mRNA (Table 1). The one exception was the lower anoxic zone (Zone 3). Although two libraries were generated, the mRNA library was lost during the sequencing process; therefore, only the results of the total RNA library are reported for Zone 3. For each of the metatranscriptomic libraries, between 32 and 39 million high quality reads were recovered, with the majority of the sequences classified as ribosomal RNA (rRNA; Table 1). The mRNA enrichment procedure reduced rRNA levels by only 15% and changed the relative taxon abundances of recovered rRNAs, particularly in Cyanobacteria and Eukaryota, compared to the total RNA (Supplementary Fig. S1a). However, the mRNA enrichment did not increase the percent of protein-encoding sequences annotated (Table 1), nor were major differences observed between the total RNA and mRNA libraries (Supplementary Fig. S1b). These results suggest that the additional mRNA enrichment process did not enhance the metatranscriptomic sequencing effort and that the two libraries were comparable.

To complement the metatranscriptomic libraries, a metagenome from the whole mat (1–9 mm) was sequenced and assembled into contigs using the procedures outlined in the materials and methods (Table 1). Of the 103 million metagenomic reads only 1.6 million reads were assembled into contigs longer than 200 bp and 61% of the predicted protein-encoded genes in the assembled metagenome were annotated. This low recovery of assembled metagenomic reads may reflect the short read length or the high diversity of organisms (>800 OTUs) found in the thrombolitic mats^24^,^47^,^49^,^53^. Additionally, the program Bowtie^54^ was used to recruit the metatranscriptomes from the three zones to the assembled metagenome contigs: Zone1 (8% total RNA, 13.17% mRNA); Zone 2 (4.14% total RNA, 6.82% mRNA); and Zone 3 (2.24% total RNA). The highest recruitment was observed in Zone1 and may reflect a higher number of previously described, and sequenced, species within the top layer of the thrombolite mat community.

### Active members of the thrombolitic mat community during midday

Taxonomic diversity in the thrombolitic mat community has been previously assessed through 16S and 18S rRNA gene surveys^24^,^47^, 18S rRNA transcript amplicon analysis^55^, and unassembled metagenome analysis^53^. In this study, community gene expression profiling was used to capture a more comprehensive picture of the actively transcribing populations (i.e., active) within the thrombolitic mats providing the first spatial distribution of taxonomic diversity within a lithifying mat community.

**Figure 2:**
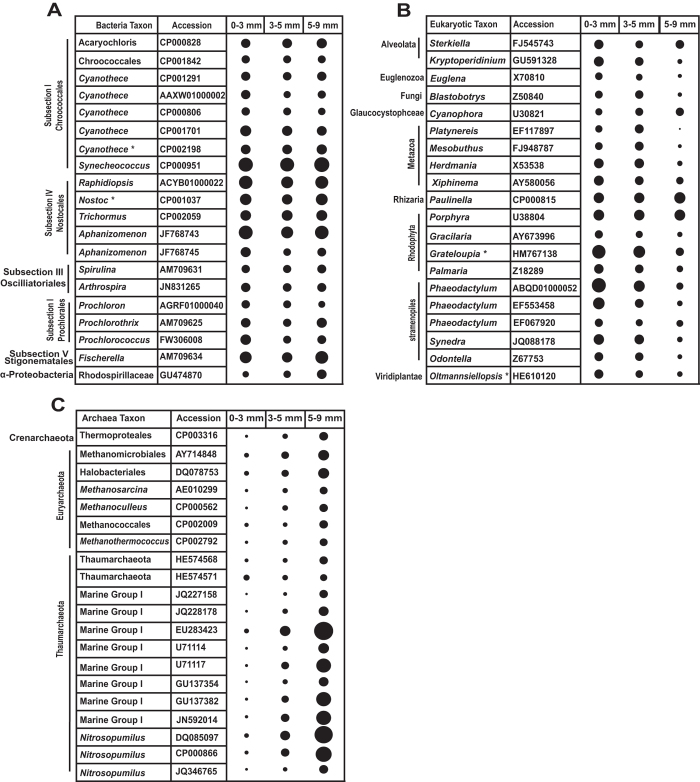
Relative abundance of the 20 most abundant taxa per domain based on expressed ribosomal RNAs from the total RNA extracts. (**a**) Bacteria, (**b**) Eukaryota and (**c**) Archaea. The SILVA taxonomic scheme was used for Bacteria and Archaea. The EMBL taxonomic scheme was used for Eukaryotes. Each domain was examined separately and the size of the circle reflects the number of rRNAs recruited to each rRNA gene from each domain. The following are the ranges for each domain from smallest number of reads represented to the largest number of reads: Bacteria (84,534–641,515); Eukaryota (86–144,214); Archaea (10–3,788). Asterisks (*) indicate the presence of a particular taxon recovered from rRNA reads in the assembled metagenome.

Taxonomic assignment of rRNAs and mRNA transcripts indicated that the Cyanobacteria and Proteobacteria were the most active phyla in the thrombolitic mats of Highborne Cay at midday (Fig. 2a and S1), supporting prior 16S rRNA gene diversity^30^,^31^,^47^ and biogeochemical analyses^23^,^24^. The relative abundance of Cyanobacteria decreased with depth, whereas the abundance of transcripts from non-cyanobacterial groups, such as Proteobacteria, Firmicutes, Bacteroidetes, and Planctomycetes increased within depth (Supplementary Fig. S1). When considering the distribution of rRNAs from the 20 most abundant bacterial taxa, the cyanobacterial orders Chroococcales, heterocystous Nostocales, and filamentous Oscillatoriales were enriched throughout the thrombolitic mat (Supplementary Fig. S1).

**Figure 3:**
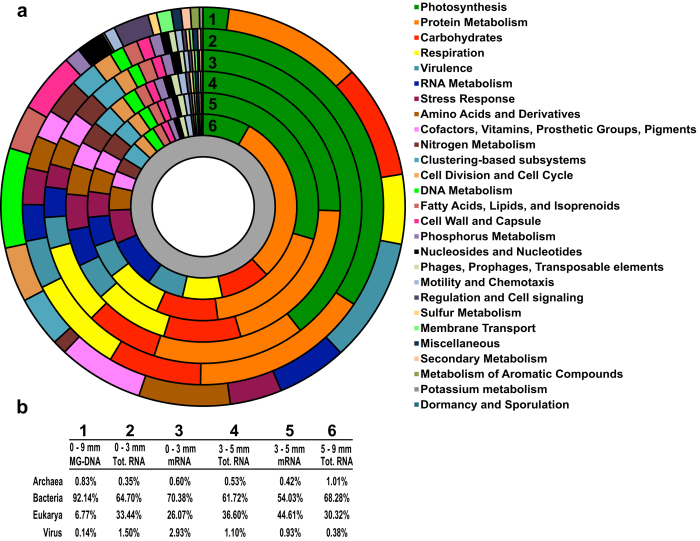
Spatial profile of functional gene expression throughout thrombolitic mat. (**a**) Relative abundance of SEED subsystems (Level 1) based on RefSeq annotations of protein-encoding reads. The subsystems in the legend are ordered based on overall abundance within the assembled metagenome (outer ring 1) and the metatranscriptomes (inner rings 2–6). (**b**) Differences in community composition based on taxonomic assignment of all RefSeq annotations for each sequenced library. Domain-level differences are given as percent of those RefSeq annotations that were assigned to a specific taxonomy.

In addition to the bacteria, eukaryotes also appear to be active members of the thrombolitic mat community^24^,^48^,^49^ (Fig. 2b and S1). Although fewer eukaryotic transcripts were recovered in each zone compared to bacteria (Fig. 3), our analysis of the rRNA expression indicated a wide range of abundant active eukaryotic transcripts that could be assigned to marine worms and arthropods (Metazoans), algae (Rhodophytes, Viridiplantae), protists (Alveolata, Ciliates), diatoms (Stramenopiles), Fungi, and amoebas and foraminifera (Rhizaria) (Supplementary Fig. S1). Within these dominant eukaryotic groups, rRNA abundances of the photosynthetic and heterotrophic organisms were enriched in the top 5 mm of the mat (Zones 1 and 2) with protists, photosynthetic amoeba, and algae having similar distribution throughout the mat profile (Fig. 2b). This distribution likely reflects the aerobic nature of these organisms due to the oxic environment. Similar eukaryotic diversity was found in a study of amplified expressed r18S rRNAs^49^ and 18S rRNA genes^24^ and the differences in relative abundances of taxa may reflect primer biases in gene marker-based studies. For example, active foraminifera have been previously documented in the top 10 mm of thrombolitic^48^,^49^, however, these organisms made up less than 1% of transcript reads within our depth profile. Together, these results suggest that eukaryotes are metabolically active in thrombolitic mat community, albeit at lower level than the bacteria.

In addition to bacteria, archaea have been identified within lithifying microbial mat communities^3^,^47^,^52^. In the thrombolite metatranscriptome, archaeal transcripts ranged from 0.35% in the oxic top 3 mm up to 1.3% in the bottom 5 mm of the mat (Fig. 2c and S1). The high abundance of ammonia-oxidizing Thaumarchaeota and halophilic Euryarchaeota in the Highborne Cay thrombolites was previously detected in a 16S rRNA gene survey^47^. Interestingly, rRNAs similar to several methanogenic archaea were also recovered; these organisms have not been previously detected in 16S rRNA gene censuses of lithifying mats^3^,^46^,^47^ even though methane production is often associated with microbial mats and microbialites^18^,^30^. Protein-encoding reads recovered from the anoxic zone indicated the presence of metabolically active methanogenic Euryarchaeota within this lower anoxic portion of the mat, however, transcripts related to this metabolism was not recovered within our metatranscriptomes (Supplementary Fig. S1b). Overall, the active bacterial, archaeal and eukaryotic populations in the thrombolitic mat community showed distinct taxonomic gradients within the thrombolitic mats. Although taxonomically distinct from neighboring stromatolites^31^,^47^, the results suggest that the mats associated with unlaminated clotted fabrics of the thrombolites also exhibit a distinctive zonation of microbes throughout the mat community. much like their stromatolite counterparts.

### Spatial gradients of functional gene expression within the thrombolitic mat

Previous studies have shown that the thrombolite mat community of Highborne Cay generates gradients of oxygen, hydrogen sulfide^49^, and carbon substrate utilization^53^ that are linked to the diel cycle, as seen in other microbialitic systems^11^. In this study, metatranscriptomic profiling was used to determine the spatial distribution of functional gene expression at midday (Fig. 3). To preserve the taxonomic affiliation of functional genes, the RefSeq annotations were processed through MEGAN resulting in between 50 to 60% of our annotated reads being assigned to both a specific taxonomy and gene function (Fig. 3b and S1b). The SEED subsystem and KEGG metabolic pathways were used for in-depth analysis of thrombolitic mat gene function. A caveat to this approach, however, is that genes can be assigned to more than one subsystem or pathway and may have potentially different functions within the thrombolite. For example, the molecular chaperone GroEL is part of both the Protein Metabolism and Virulence subsystems, leading to the number of reads assigned to pathways and subsystems not being a one to one ratio with the number of reads annotated.

Within the thrombolitic mat metatranscriptomes, most of the annotated functional gene transcripts were bacterial (61.7–68.3%) in origin, whereas the remaining were either eukaryotic (30.3–33.4%), archaeal (0.4–1%), or viral (0.4–1.5%) (Fig. 3b). Most eukaryotic transcripts were either hypothetical proteins or could not be assigned to a subsystem, and only 1–2% of the remaining eukaryotic functional genes reads were annotated. These transcripts were primarily respiration and protein synthesis genes, suggesting that heterotrophic eukaryotes contribute to mat metabolic cycling through consumption of bacterial biomass^48^ (Supplementary Fig. S1b and S2). Based on the protein-encoding genes, archaea made up an increasing percent of the metatranscriptomic libraries with depth, suggesting that they are an active component of the deeper mat community (Fig. 3). Compared to the eukaryotic transcripts, the archaeal mRNA transcripts were metabolically diverse, suggesting that archaea may be involved in a wide range of metabolic activities within the thrombolitic mat, such as nitrogen fixation and sulfate reduction (Supplementary Fig. S2). Viral proteins transcripts were recovered throughout the mat with the highest concentration in the top 3 mm (Zone 1). Most of these reads were similar to proteins from cyanophage, including potentially cyanophage-derived *psbA* and *psbD* gene transcripts, indicating that active cyanophage infection may be occurring in the mat. It has been suggested that during infection photosystem II from *Prochlorococcus* and *Synechoccocus* phages may be supplying energy to their host cyanobacteria^56^,^57^. These results differed slightly from previous sequencing of the virome of Highborne Cay thrombolites in which the dominant recovered phage were associated with Proteobacteria^50^. The role of viruses in lithifying microbial mat communities are unclear; however, as the virome of Highborne Cay microbialites has been shown to be highly divergent from that of the global virome^50^. Viruses, in particular phages, may be playing a key role in growth rates, adaptations and genetic exchange throughout the thrombolite mat community.

### Cyanobacteria metabolisms dominate thrombolite community at midday

**Figure 4:**
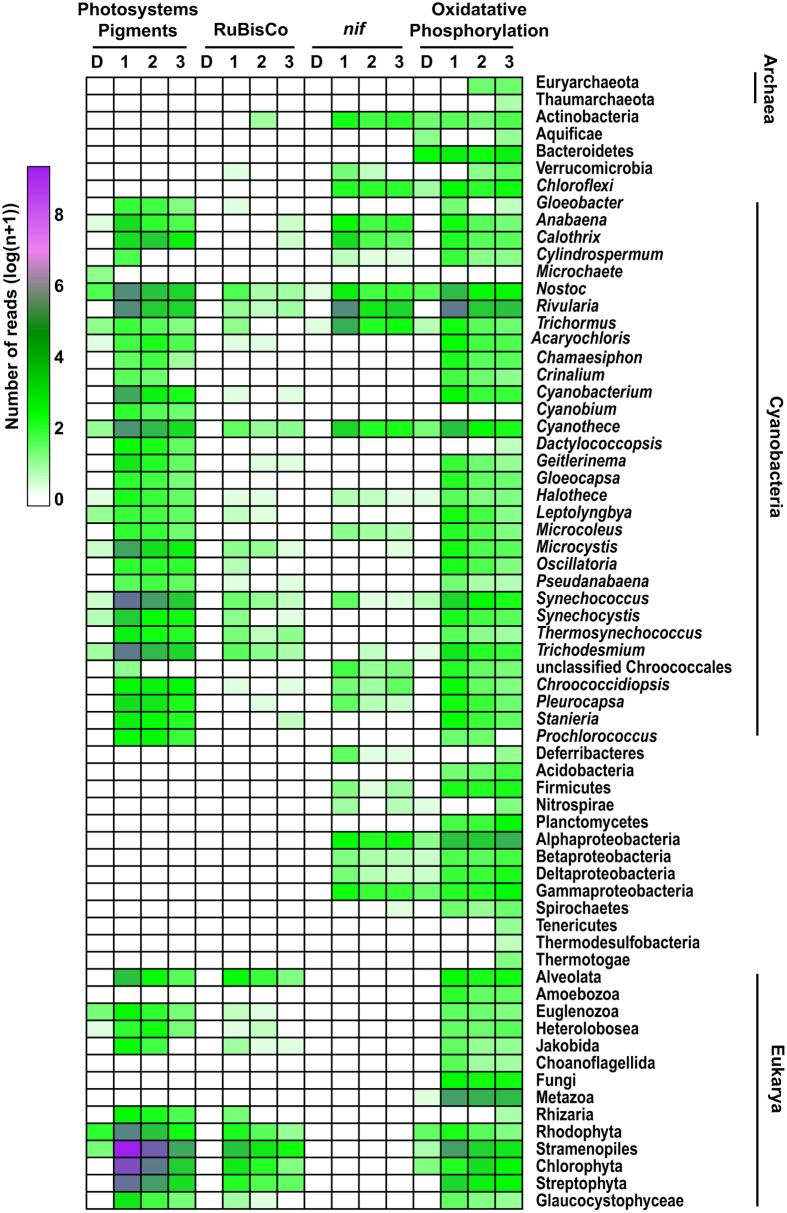
Organisms involved in energy transformations within the thrombolitic mat during midday. Functional annotation was carried out on the RefSeq annotations from the assembled metagenome (D), Zone 1 (1), Zone 2 (2), and Zone 3 (3) using the KEGG pathways database. Read abundance was normalized by log(n + 1) scaled to the number of reads for each dataset. Photosystem genes and antenna-pigments represent oxygenic photosynthesis. Ribulose-1,5-bisphosphate carboxylase oxygenase (RuBisCO) genes were used as proxies for bacterial and eukaryotic carbon fixation. Nitrogenase (*nif*  ) genes were used to represent nitrogen fixation occurring within the thrombolitic mat. Genes involved in oxidative phosphorylation were used as a proxy for respiration. Genes with high relative abundance appear purple while those at lower abundances are green.

Although the recovered photosynthesis transcripts only made up 2% of the metagenomic contigs, they represented greater than 34% of the transcripts in the top two zones of the mat and 8% of the reads in the bottom anoxic zone, suggesting oxygenic photosynthesis was the most active metabolism in the upper regions of the mat and that metagenomic analyses alone do not accurately reflect the interactive network and activities of the microbial community (Fig. 3). Additionally, there was a wide taxonomic distribution of cyanobacterial photosystem genes and pigments transcripts throughout the thrombolitic mat (Fig. 4; Supplemental Table S1). Most of these recovered transcripts were closely related to the order Nostocales specifically the genera *Nostoc* spp., *Calothrix* spp. *Rivularia* spp. (Fig. 4). All three of these taxa are closely related to the unsequenced *Dichothrix* sp., the dominant cyanobacteria observed in both 16S rRNA^47^, microscopic^23^,^24^,^47^ and petrographic thin section analyses^23^,^24^ (Fig. 1e). Efforts to sequence *Dichothrix* sp. are currently underway (Foster unpublished). Contrastingly, very few bacterial reaction center transcripts for anoxygenic photosynthesis from purple non-sulfur bacteria were recovered, suggesting these taxa have low phototrophic transcriptional activity at midday.

The high number of cyanobacterial photosynthetic gene transcripts (Figs 3 and 4) not only reinforces previous biogeochemical observations that oxygenic photosynthesis is a dominant metabolism in the thrombolite mat community, but the results, more importantly, help to delineate the specific taxa and genetic pathways that are active and may be altering the microenvironment of the mat community to drive an increase in the pH thereby promoting carbonate precipitation^10^,^23^,^24^,^53^. Elevated rates of photosynthetic CO_2_ uptake can result in a depletion of carbon dioxide (CO_2_), thereby altering the carbonate equilibrium (HCO_3_^−^ ↔ CO_2_ + OH^−^) such that bicarbonate disassociates to carbon dioxide and a hydroxyl anion^10^,^18^. Therefore, the high rates of photosynthesis coupled with the fixation of CO_2_ in the upper portions of the thrombolite mat during midday may be rapidly changing the saturation index of the local environment thereby promoting carbonate precipitation.

Although cyanobacteria were the dominant taxa fixing carbon, analysis of the recovered 1,5-ribulose bisphosphate carboxylase oxygenase (RuBisCO) gene transcripts from the thrombolite spatial metatranscriptome profiles indicated that in addition to the cyanobacteria, there was a wide range of bacteria and eukaryotes actively fixing inorganic carbon through the Calvin-Benson-Bassham cycle (Fig. 4). Of these different taxa, the Alphaproteobacteria (e.g., Rhizobiales) produced the highest number of transcripts in upper oxic zone (Fig. 4 and S3). The Calvin-Benson-Bassham cycle is a functional carbon fixation pathway in several bacterial anoxygenic phototrophs and chemolithoautotrophs^58^, which have previously been identified as prominent members of the thrombolitic mat community^47^,^53^.

### Expression of nitrogen fixation genes throughout the thrombolitic mat at midday

In addition to photosynthesis and carbon fixation, the thrombolite metatranscriptome revealed that nitrogen fixation was also an active metabolism at midday and identified those taxa associated with the metabolism (Fig. 3). All organisms require nitrogen for growth and in the low-nutrient oligotrophic waters where the Bahamian thrombolites are found biological nitrogen fixation is a critical pathway for the mat ecosystem to provide nitrogen for growth of the community^29^,^43^,^59^. The process is catalyzed by the enzyme nitrogenase, which is a ubiquitous, highly conserved enzyme found in a broad range of microbes^60^,^61^. In the modern thrombolites of Highborne Cay, a diverse consortium of putative diazotrophic organisms was active throughout the spatial profile of the mat communities. The diversity of bacterial *nif* transcripts (e.g., *nif D*, *nif H*, *nif K*) detected in the metatranscriptomes was higher than previously observed in *nif H* gene surveys of microbialites^29^,^43^,^62^ and in the thrombolite metagenome^53^ (Fig. 4; Supplemental Table S1). Bacterial *nif* transcripts were well represented throughout the three thrombolitic mat zones, comprising nearly 5% of the annotated reads in upper Zone 1 and 3% in Zones 2 and 3 and were dominated by Cyanobacteria (Fig. 4). In the upper zone of the thrombolitic mat community there was an enrichment of *nif* transcripts derived from heterocyst-forming cyanobacteria capable of nitrogen fixation in the presence of oxygen with sequence similarity to several genera within the order Nostocales including *Rivularia*, *Anabaena*, *Calothrix*, and *Nostoc* (Fig. 4). Additionally, expression of *nif* genes by non-heterocystous cyanobacteria were detected throughout the thrombolitic mat, but at much lower levels with similarity to *Cyanothece*, *Halothece*, *Microcoleus*, *Synechococcus*, *Pleurocapsa* and several unclassified Chroococcales (Fig. 4).

In addition to the cyanobacteria, *nif* transcripts were also recovered from several other taxa throughout the mat including Actinobacteria, Chloroflexi, Alphaproteobacteria, Gammaproteobacteria and to a lesser extent Verrucomicrobia, Firmicutes, Betaproteobacteria, Deltaproteobacteria and Deferribacteres (Fig. 4). The levels of expression in thrombolitic mats at midday correspond to those in other lithifying microbial mat systems derived from Alchichica crater-lake, Muyil costal lagoon, and Cuatro Ciénegas in Mexico^43^,^62^. Together the results suggest that thrombolites rely on a diverse assemblage of nitrogen-fixing microbes with a wide range of metabolic strategies to facilitate the influx of nitrogen into the thrombolitic mat community and potentially providing an important mechanism for nutrient cycling within the thrombolitic mats.

### Respiration transcripts in thrombolitic mat at midday

Respiratory processes in microbialite systems play a key role in the microbially mediated carbon cycle via the consumption of photosynthesis-derived biomass, which has the potential to release calcium from the EPS matrix and can subsequently influence carbonate precipitation^11^,^20^,^23^,^24^,^28^,^30^,^32^,^33^. The expression of genes involved in oxidative phosphorylation, including dehydrogenases, cytochrome oxidases, and ATPases were used as proxies, and indicated a diverse community at midday that decreased in relative abundance with depth (Fig. 4; Supplemental Table S1). Bacteria comprised between 83–88% of the total respiration transcripts with Actinobacteria, Cyanobacteria, Firmicutes, Alphaproteobacteria, and Gammaproteobacteria having the proportion of recovered transcripts (Fig. 4). Eukaryotes comprised between 11–16% of the respiration reads, whereas archaea accounted for only 1–1.4%. Few transcripts associated with anaerobic respiration processes, such as sulfate reduction (i.e., ATP sulfurylase, APS reductase, sulfite reductase and thiosulfate reductase), denitrification, and methanogenesis were recovered in the midday metatranscriptomic libraries, suggesting these key metabolisms, known to be active in both thrombolites and stromatolites^18^,^19^,^32^,^53^, may be overshadowed by photosynthetic pathways or have a rapid transcript turn over. Additional time points are required to fully assess the activity and expression of these anaerobic metabolisms throughout the thrombolitic mat community.

### Expression of genes associated with cyanobacterial exopolymeric substances

**Figure 5:**
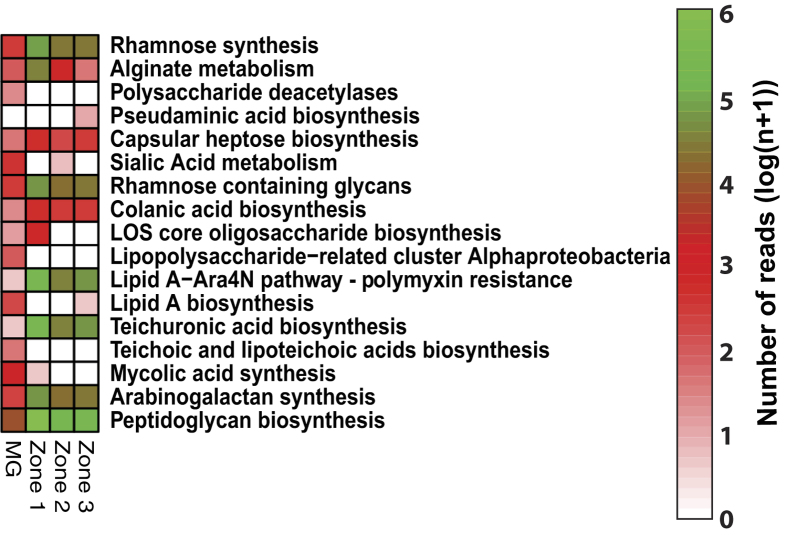
Expression of genes associated with exopolymeric substance (EPS) production. Heat map comparing the relative abundance of functional gene categories associated with the Cell Wall and Capsule SEED subsystem from the assembled metagenome (MG) and the metatranscriptomes (Zone 1–3). Read abundance for the metagenome and metatranscriptomes were separately normalized by log(n + 1) and scaled to the number of reads for each dataset. Those genes that are highly abundant appear green with those that are at lower abundances, within the recovered libraries are in red.

In addition to the structural and protective roles that exopolymeric substances (EPS) play in lithifying microbial ecosystems, the EPS matrix strongly binds cations and serves as nucleation sites for calcium carbonate precipitation^10^,^33^,^36^,^63^. Although numerous genes associated with exopolysaccharide production were observed in the metagenome, the metatranscriptome analyses of the three mat zones revealed differential expression of these exopolysaccharide-associated genes (Fig. 5). In the upper 3 mm of the thrombolitic mat there was an increase in the number of transcripts associated with alginate, rhamnose synthesis, and rhamnose-containing glycans primarily derived from the cyanobacterial order Nostocales (Fig. 5). Similar pathways have been observed in the metagenomes of other cyanobacterial-dominated microbialites derived from Cuatro Ciénegas^4^. Alginate and rhamnose-containing glycans have been shown to contribute to the hydrophobicity of EPS material and can contribute to the rheological properties (e.g., viscosity) of the EPS material^64^,^65^ but are unlikely to be the labile portion of the EPS matrix and may not have a rapid turnover. Other genes that were in high abundance in the upper 3 mm of the thrombolites were associated with uronic acid biosynthesis (Fig. 5). One relatively unique feature in cyanobacterial EPS is the high abundance of uronic acids, which help to confer a negative charge to the EPS material and increases its affinity for the adsorption of cations, such as Ca^2+^^36^,^65^. The recovered transcripts associated with the production of these molecules shared the highest sequence similarity to the cyanobacterial order Nostocales and previous studies have shown that uronic acids are highly labile to microbial mat isolates^34^,^66^. Additionally, cyanobacterial EPS derived from Bahamian stromatolitic mats has been shown to be approximately 50% mono-, di- and polysaccharides^67^. Sugar synthesis genes (i.e. mono-, di-, polysaccharides) transcripts from Cyanobacteria, Alpha- and Gammaproteobacteria, and Chloroflexi were found in all three zones; however, the metabolism of certain hexoses (e.g., mannose) and pentoses (e.g., xylose, fucose) previously shown to be abundant in stromatolite EPS^68^ were enriched in the upper zone of the thrombolite mat. In the lower zones of the mat (3–9 mm) there were fewer cyanobacterial transcripts and an increase in proteobacterial transcripts of genes more typical of cell wall and envelope synthesis, such as colonic acid, lipopolysaccharide (i.e., lipid A) and peptidoglycan (Fig. 5).

In addition to EPS production genes, spatial differences were observed in sugar reduction metabolisms that could be associated with EPS degradation and turnover (Supplementary Fig. S3). Although EPS turnover is a complex process within the mats, the alteration and restructuring of the EPS material through microbial metabolisms, including but not limited to sugar degradation, can free cations into the local environment, thereby facilitating the precipitation of carbonate within the mat community^10^. Previous studies, using hydrolytic enzymes (e.g., α-, β-glucosidase and β-galactosidase) as proxies for EPS degradation, have shown pronounced gradients of reductase activity with depth in microbial mats^66^. As in these previous studies, the total number of transcripts involved in sugar reduction decreased with depth in the thrombolitic mat but there were spatial differences in relative abundances of different sugar metabolism subsystems (Supplementary Fig. S3a). There was a high abundance of transcripts associated with mannose and galactose degradation in the upper 3 mm of the thrombolites, whereas transcripts for genes involved with organic acid and alcohol metabolism from non-cyanobacterial phyla were enriched in the deeper anoxic zone 3 (Supplementary Fig. S3). These findings are consistent with metagenomic and substrate utilization experiments in the adjacent stromatolites that indicate spatial differences in heterotrophic sugar utilization and degradation are present^18^,^34^,^52^,^53^.

### Comparison of thrombolite metatranscriptome to other microbialite communities

**Figure 6:**
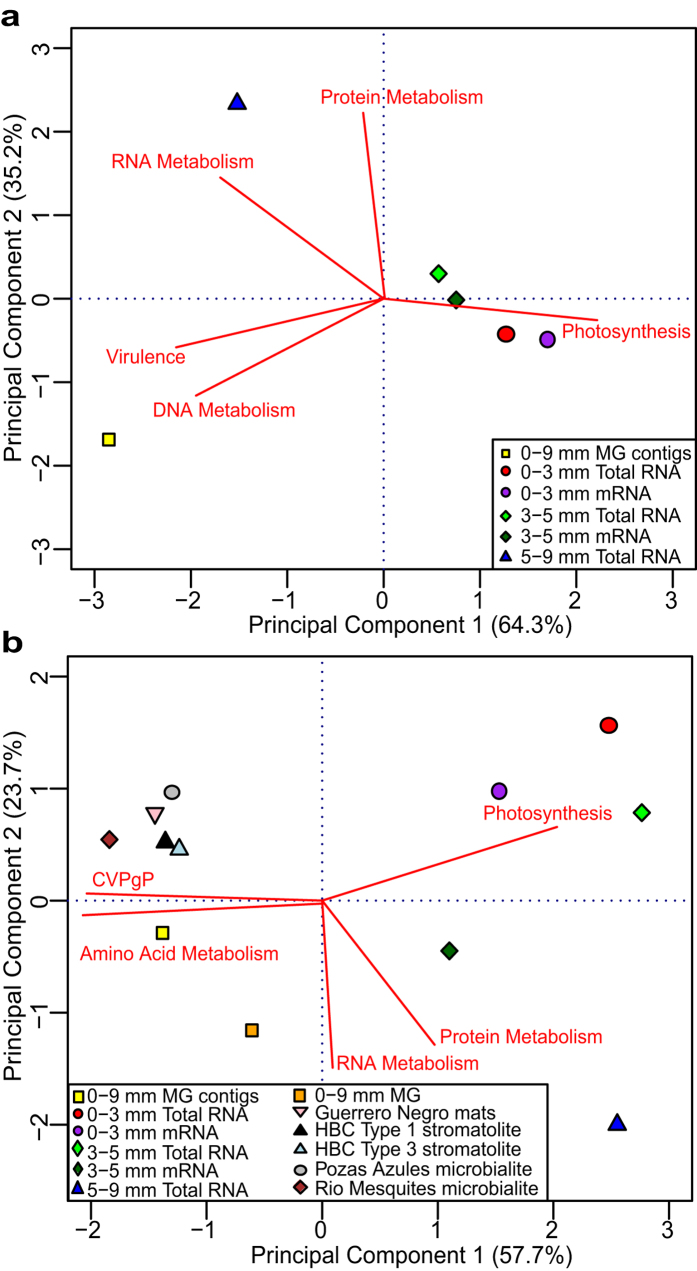
Principal coordinates analysis of functional gene abundances of thrombolitic mat metatranscriptomes based on SEED subsystems (Level 1). The red biplot lines represent the top five subsystems explaining the differences between the samples included in the analysis. (**a**) Comparison of thrombolitic mat metatranscriptomes and assembled metagenome from this study (**b**) Comparison to metagenomes of previously sequenced lithifying and non-lithifying microbial mat communities based on MG-RAST annotation (e-value 10^−5^). CVPgP represent the Cofactors, Vitamins, Prosthetic Groups, Pigments subsystem.

As this study represents the first analysis of the metatranscriptome from a lithifying microbial mat community, direct comparisons with equivalent systems were not possible. However, to further understand the metabolic variation between the different spatial metatranscriptomes, a principal component analysis of the assembled metagenome, total RNA and mRNA libraries was conducted (Fig. 6a). The analysis revealed that 99% of the variation between libraries could be explained by five metabolic categories: Photosy-nthesis, Protein Metabolism, RNA metabolism, Virulence, and DNA Metabolism (Fig. 6a). Thrombolite metagenomic contigs did not cluster tightly with the metatranscriptomic libraries, indicating differences in the subsystems abundances. For example, Cell division and Cell Cycle, and Phosphorus, Potassium, and Sulfur metabolism subsystems comprised a higher proportion of the assembled metagenome. This outcome may have resulted from the assembly capturing dominant metabolisms from those abundant organisms within the thrombolitic mat, as opposed to representing full metabolic potential of the whole community, as the unassembled reads do.

Additionally, the thrombolite metatranscriptome and partially assembled metagenome were compared to other lithifying and nonlithifying microbial mat ecosystems to assess those shared metabolisms and subsystems (Fig. 6b). Metagenomic data from the following ecosystems were also included (see methods section for study ID numbers): unassembled thrombolite metagenome from Highborne Cay, The Bahamas^53^; non-lithifying (Type 1) and lithifying (Type 3) stromatolites from Highborne Cay^52^; freshwater microbialites from Pozas Azules and Rio Mesquites, Cuatro Ciénegas, Mexico^4^,^69^,^70^; and hypersaline non-lithifying mats from Guerrero Negro, Mexico^71^. When comparing the thrombolite metagenome and metatranscriptome to the metagenomes of other types of microbialites, the thrombolites were distinct (Fig. 6b). For example at Highborne Cay stromatolites and thrombolites are juxtaposed within a few meters, yet the metagenomes of these two communities clustered separately, suggesting metabolically distinct communities are associated with these two types of microbialites. This result is also supported by previous comparisons of oxygen productivity where the Highborne Cay thrombolites had 2–5x higher production rates than the adjacent stromatolites^24^. Interestingly, the Highborne Cay stromatolites shared more sequence similarity to the freshwater microbialites Cuatro Ciénegas and the nonlithifying hypersaline mats of Guerrero Negro in Mexico than the Highborne Cay thrombolites, and both systems had lower abundances of vitamin biosynthetic pathways as part of the Cofactor, Vitamin, Prosthetic Groups and Pigment subsystem (Fig. 6b). These results indicated that although many pathways were shared between thrombolites and other microbialite ecosystems, the thrombolite microbiome harbors a distinctive set of microbial processes that separates it from other lithifying and nonlithifying mat ecosystems and may potentially contribute to the clotted microfabric characteristic of modern thrombolites. Alternatively, this difference could be the result of an artifact of sequencing technology, as all the microbial mat metagenomes used in this comparison were previously sequenced using Sanger shotgun libraries or 454 pyrosequencing, rather than the Illumina GAII platform in this study.

Taken together, in this study we gained a new perspective of thrombolite community dynamics and how the active community members are spatially organized at the molecular level. The metatranscriptomic analyses of the thrombolites revealed the metabolically active pathways that form discrete spatial gradients throughout the thrombolite mat communities at midday. This work can serve as the foundation for future analyses to examine temporal changes in the metabolic activity throughout the diel cycle as well as in response to seasonal environmental changes, such as photosynthetic active radiation levels, temperature, and wave energetics that have been previously observed at Highborne Cay^72^. Comparative metagenomics also revealed that, despite several shared metabolisms, differences were observed between the microbialite metagenomes even in those laminated stromatolites located only a few meters away from the thrombolites. These findings suggest that the molecular differences observed between the communities may be influencing the formation of the distinctive microfabrics of the microbialite deposits. Taken together, these new molecular insights into the modern thrombolite community can potentially help delineate those conserved core metabolisms and taxa essential for biologically induced precipitation thereby providing an important tool for understanding microbe-mineral interactions.

## Methods

### Sample collection

Thrombolitic mats were collected from the island of Highborne Cay, The Bahamas (76°49’ W, 24°43’ N) in February and March 2010. Samples for the metatranscriptomes (n = 3) were collected at midday (i.e., 12–12:30 pm) from a single intertidal thrombolite platform from Site 5, as designated by Andres and Reid^73^, sectioned into three horizontal zones (0–3 mm, 3–5 mm and 5–9 mm), based on microelectrode depth profiles (see below) and immediately placed in RNALater (Life Technologies, Inc., Grand Island, NY), resulting in three biological replicates for each zone. Corresponding whole, un-sectioned mat samples were collected from the same platform for metagenomic analysis (n = 3) and petrographic thin sectioning (n = 3). For petrographic thin section analysis samples were dried at 60 °C for 24 h. The samples were cast in Buehler EpoxiCure resin and hardened samples were mounted to glass and cut down to 30 μm thin sections. Areas of interest (≈5 mm) were selected using an Olympus BH2 petrographic microscope.

### Microelectrode depth profiles

Prior to sample collection, biochemical depth profiles for oxygen were measured *in situ* within the thrombolitic mats. Mats, covered by up to 15 cm of water were probed using polarographic Clark-type needle electrodes with an outer diameter of 0.4 mm and a sensing tip of 30–60 μm. Measurements were taken in 200 μm increments with the aid of a hand-operated micromanipulator (M3301; World Precision Instruments, Sarasota, FL). The signal was recorded with a picoammeter (PA2000; Unisense, Aarhus, Denmark).

### Nucleic acid extraction and purification

Total RNA was extracted from each thrombolitic mat zone biological replicate for total RNA (n = 3) and mRNA-enriched RNA (n = 3) by a modified RNAzol protocol (MRC, Cincinnati, OH). Briefly, 1 g of mat was ground to a fine powder in a mortar under liquid N_2_ then divided into 80–100 mg aliquots that were each added to 1 mL RNAzol RT reagent. These mixtures were vortexed for 15 s and centrifuged for 5 min at 23 °C. The supernatants were transferred to fresh tubes and DEPC-treated water was added to precipitate the DNA and proteins. After mixing through inversion, the samples were incubated for 15 min at 23 °C, then centrifuged for 15 min at 4 °C to recover the RNA-containing supernatant. To increase recovery of total RNA, 1 μl of RNA precipitate carrier (MRC, Cincinnati, OH) was added prior to precipitation with 1 mL 75% ethanol. These samples were incubated at 23 °C for 10 min followed by centrifugation for 8 min at 4 °C. The resulting total RNA pellets were washed twice with 75% ethanol, air dried for 2 min, then resuspended in molecular grade water. The aliquots from each biological replicate were pooled prior to further processing resulting in three separate RNA extractions for each zone.

All RNA samples were treated with Turbo-DNase (Life Technologies, Inc., Grand Island, NY) to remove any remaining DNA and re-concentrated with the MegaClear kit (Life Technologies, Inc., Grand Island, NY). Any additional inhibitors were removed with a lithium chloride precipitation step, as described in manufacturer’s protocol (Life Technologies, Inc., Grand Island, NY). To generate the mRNA-enriched fractions, total RNA (2 μg) was enriched for mRNA using MicrobExpress (Life Technologies, Inc., Grand Island, NY), which removed rRNAs from the samples. This enrichment was followed by an additional purification with the MegaClear kit.

Message AMP II-Bacterial RNA kit (Life Technologies, Inc, Grand Island, NY) was used to amplify the total RNA (40 ng) and mRNA-enriched fractions (20 ng) from each biological replicate from the mat zones (n = 3). The *in vitro* transcription reaction was incubated for 14 h at 37 °C and the resultant antisense RNA was purified and concentrated. cDNA was synthesized from the 1 μg amplified RNA reaction product in triplicate using Promega Universal RiboClone cDNA Synthesis System (Promega, Madison, WI) with random hexamer primers and 1.25 U GoScript enzyme (Promega, Madison, WI). The second strand synthesis step was performed with T4 polymerase using manufacturer’s protocol. The double stranded cDNA was purified with the Wizard Genomic DNA Purification kit (Promega, Madison, WI) and 1 μg of cDNA from the each of the biological replicates from each mat zone were pooled prior to sequencing. To complement the metatranscriptomic libraries, metagenomic DNA was extracted from corresponding whole thrombolitic mat sections (0–9 mm; n = 3) using a previously described xanthogenate method that has been optimized for microbialites^45^.

### DNA sequencing and read processing

DNA and cDNA were sequenced using the Illumina GAIIx platform at the University of Florida’s Interdisciplinary Center for Biotechnology Research generating paired-end reads each of 108 bp in length. Raw sequencing data is available at NCBI Bioproject PRJNA261361. All Illumina libraries were trimmed and quality filtered with Prinseq to remove reads with ambiguous positions, quality scores below 20, and low complexity sequences^74^. Quality-filtered reads were analyzed as described below. Separate assemblies of the metagenomic reads were performed using a hybrid assembly approach as described^75^,^76^. These previous studies have shown that one can obtain improved assemblies from uneven Illumina metagenomic datasets by using several different de novo assembly protocols prior to a final assembly. Briefly, the metagenomic reads (n = 3) underwent 18 *de novo* assemblies (3.2 million contigs) using different kmer (K) sizes with the programs MetaVelvet (K = 21, 25, 29) and SOAPdenovo (K = 23, 27, 31). The resulting contigs from the three metagenomes were merged and underwent a final assembly with Newbler (version 2.7; Roche, Branford, CT), where 1.7 million of these contigs were used to make the final contig set (90,463) with an average quality score of 63.3 with the following parameters: -ml 100 -mi 95 −a 200 −l 500. Ribosomal RNA genes were identified and analyzed from the assembled contigs as described below. Protein-encoding genes in the contigs were identified using the program MetaGeneMark^77^ and analyzed with the same gene annotation process as the messenger RNA as described below.

### Ribosomal RNA transcript analysis and classification

Ribosomal RNAs were identified and removed from the metatranscriptomic libraries by blastn (maximum e-value 1e^−5^) against the combined SILVA small subunit and large subunit databases (V-111). Blastn hits to rRNA genes from land plants (Embryophytes) were excluded from this analysis. Following annotation, read counts were parsed using SILVA mapping files with SILVA (Archaea, Bacteria) and EMBL (Eukaryota) taxonomy assignments. For the total RNA libraries, Phyloseq^78^ was used for comparative analyses of the different thrombolitic mat zones. Bubble charts were generated using a previously published perl script^79^.

### Messenger RNA analysis

After removal of rRNAs, protein-encoding genes in each of the metatranscriptomes were identified with FragGeneScan assuming an error rate of one percent^80^. To assign a putative function, protein-encoded genes were annotated via BLAT against the non-redundant database protein database (nr release February 02, 2013; minimum identity 30%), with the best RefSeq hit retained (maximum score e-value 1e^−3^) to represent each annotated gene. The BLAT results were imported into MEGAN5 (version 5.0.78)^81^ for further functional analysis. To obtain a broad functional overview of each zone within the thrombolitic mat, annotated gene reads were compared to the SEED subsystem database. The complete RefSeq results for each of these reads were extracted from the BLAT results and the taxonomy assigned based on MEGAN LCA analysis. For more in-depth analyses of energy metabolisms, the KEGG pathway database was used to assign reads to protein-encoding genes.

To account for differences in sampling depth across the metatranscriptomes a normalization protocol^82^ was performed by dividing each gene count for a metabolic pathway by the total non-ribosomal read count of the total RNA datasets from each zone (Table 1) and multiplied by the mean of the total non-ribosomal read count across the three zones (9,096,066). The resulting taxonomic count data was log(n + 1) transformed in R prior to heatmap generation using the pheatmap package (version 0.7.4). To account for potential annotation deficiencies in the SEED and KEGG subsystems, the best blat hit results GenBank (GI number) from each library were searched against RefSeq records accessed on August 1, 2013 for the following genes and/or pathways: sulfate reduction (*dsrAB*), sulfur oxidation (*sox*), ammonia oxidation (*amo*), methanogenesis (formylmethanofuran dehydrogenase), and cyanophage photosystem genes (*psbD*).

### Principal component analysis of thrombolite metatranscriptome and microbialite metagenomes

The assembled metagenome contigs and raw metatranscriptomic libraries were also submitted and analyzed through the MG-RAST standard pipeline (MG-RAST version 3.3.6). Previously published microbial mat metagenome datasets were recovered from MG-RAST and compared to the results of the current study (Project number mgp6163). These datasets (study reference and MG-RAST IDs given) included: unassembled marine thrombolitic mat metagenome from Highborne Cay, The Bahamas^53^ (4513715.3, 4513716.3, 4513717.3); non-lithifying (Type 1) and lithifying (Type 3) marine stromatolitic mats from Highborne Cay, The Bahamas^52^ (4449590.3, 4449591.3); freshwater microbialites from Cuatro Ciénegas, Mexico^4^ (44440060.4, 4440067.3), hypersaline non-lithifying mats from Guerrero Negro, Mexico^71^ (4440964.3–4440972.3 were combined into one library). All samples were analyzed at a max e-value cutoff of 10^−5^ identity >60%, and minimum alignment of 45 amino acids. Differences in sampling sizes SEED subsystem (Level 1) abundance counts were normalized to total number of SEED annotated reads for each sample. To maximize high-level functional differences between the environments analyzed, Clustering-based subsystems and Miscellaneous were excluded. Principal components analyses were carried out using the R package bpca.

## Additional Information

**How to cite this article**: Mobberley, J. M. *et al*. Inner workings of thrombolites: spatial gradients of metabolic activity as revealed by metatranscriptome profiling. *Sci. Rep*. **5**, 12601; doi: 10.1038/srep12601 (2015).

## Supplementary Material

Supplementary Information

## Figures and Tables

**Table 1:** Summary of metagenomic and metatranscriptomic sequencing analysis.

Sample	Depth profile (mm)	Sequence reads^b^	rRNA gene reads^c^ (%)	Non rRNA gene reads (%)	No. protein-encoding genes^d^ (%)	Annotated proteins^e^ (% of predicted)
DNA contigs^a^	0–9.0	1590080	127 (<1.0)	90343 (>99.0)	221365 (n/a)	134662 (60.8)
Zone 1: total RNA	0–3.0	33014701	24424465 (73.8)	8590236 (26.2)	6493754 (19.6)	1001850 (15.4)
Zone 1: mRNA	0–3.0	32275041	20333226 (63.9)	11941815 (36.1)	9087212 (28.6)	1379396 (15.2)
Zone 2: total RNA	3.0–5.0	32242043	24020842 (77.0)	8221201 (23.0)	5941954 (19.0)	637344 (10.7)
Zone 2: mRNA	3.0–5.0	38667700	23842465 (61.6)	14825235 (38.4)	10656793 (27.5)	1099843 (10.3)
Zone 3: total RNA	5.0–9.0	38435679	27958917 (73.0)	10476762 (27.0)	8049230 (21.0)	1108969 (13.8)

^a^Metagenomic contigs assembled from paired-end Illumina sequences.

^b^Number of Illumina reads retained after quality filtering with Prinseq.

^c^Number of ribosomal reads identified by blastn (evalue < 10^−5^).

^d^Number of protein encoding genes predicted by FragGeneScan.

^e^Proteins annotated by BLAT against the NCBI non-redundant database (evalue < 10^−3^).
