# Supplementary material for: Inner workings of thrombolites: spatial gradients of metabolic activity as revealed by metatranscriptome profiling
Source: Sci Rep. 2015 Jul 27;5:12601. doi: 10.1038/srep12601 (PMC4515876; doi:10.1038/srep12601)
Supplement: Supplementary Information [file srep12601-s1.pdf]

Supplemental Figures and Legends:

Inner workings of thrombolites: spatial gradients of metabolic activity as revealed by metatranscriptome profiling

J.M. Mobberley[1], C. L. M. Khodadad [1], P.T. Visscher [2], R. P. Reid[3], P. Hagan[3], J.S. Foster[1*].

Supplementary Figure S1. Relative abundance of dominant taxa within the thrombolitic mat metagenome and metatranscriptomes. Dominant taxa were those phyla that comprised at least 1% of the total number of annotated reads in each domain. Those phyla that made up less than 1% were grouped into "other" for each respective Domain. (a) rRNA fraction classified based on similarity to SILVA version 111 large subunit and small subunit libraries (Bacteria and Archaea) and EMBL taxonomy (Eukarya). Due to their high abundances across the samples, Cyanobacteria were classified at the order level and Proteobacteria were classified at the class level. (b) Protein-encoding fraction assigned by MEGAN 5 based RefSeq taxonomy.

Supplementary Figure S2. Relative abundance of eukaryotic and archaeal SEED subsystem reads in the assembled metagenome and metatranscriptomes. (a) Number of reads classified as Eukarya: 0-9 mm MG- DNA (89); 0 - 3 mm Total RNA (1190); 3 - 5 mm Total RNA (661); 5 - 9 mm Total RNA (451). (b) Number of reads classified as Archaea: 0-9 mm MG-DNA (82); 0 - 3 mm Total RNA (732); 3 - 5 mm Total RNA (366); 5 - 9 mm Total RNA (806).

Supplementary Figure S3. Relative abundance of SEED carbohydrate subsystem reads associated with sugar metabolism in the assembled metagenome and metatranscriptomes. (a) Breakdown of SEED carbohydrate subsystem (Level 2). Number of reads annotated as genes involved in sugar metabolism: 0 - 9 mm MG-DNA (232); 0-3 mm Total RNA (879); 3 - 5 mm Total RNA (456); 5 -9 mm Total RNA (678). (b) Taxa relative abundance of sugar metabolism reads. Due to their high abundances across the samples, Cyanobacteria were classified at the order level and Proteobacteria were classified at the class level.

Supplemental Fig. S1

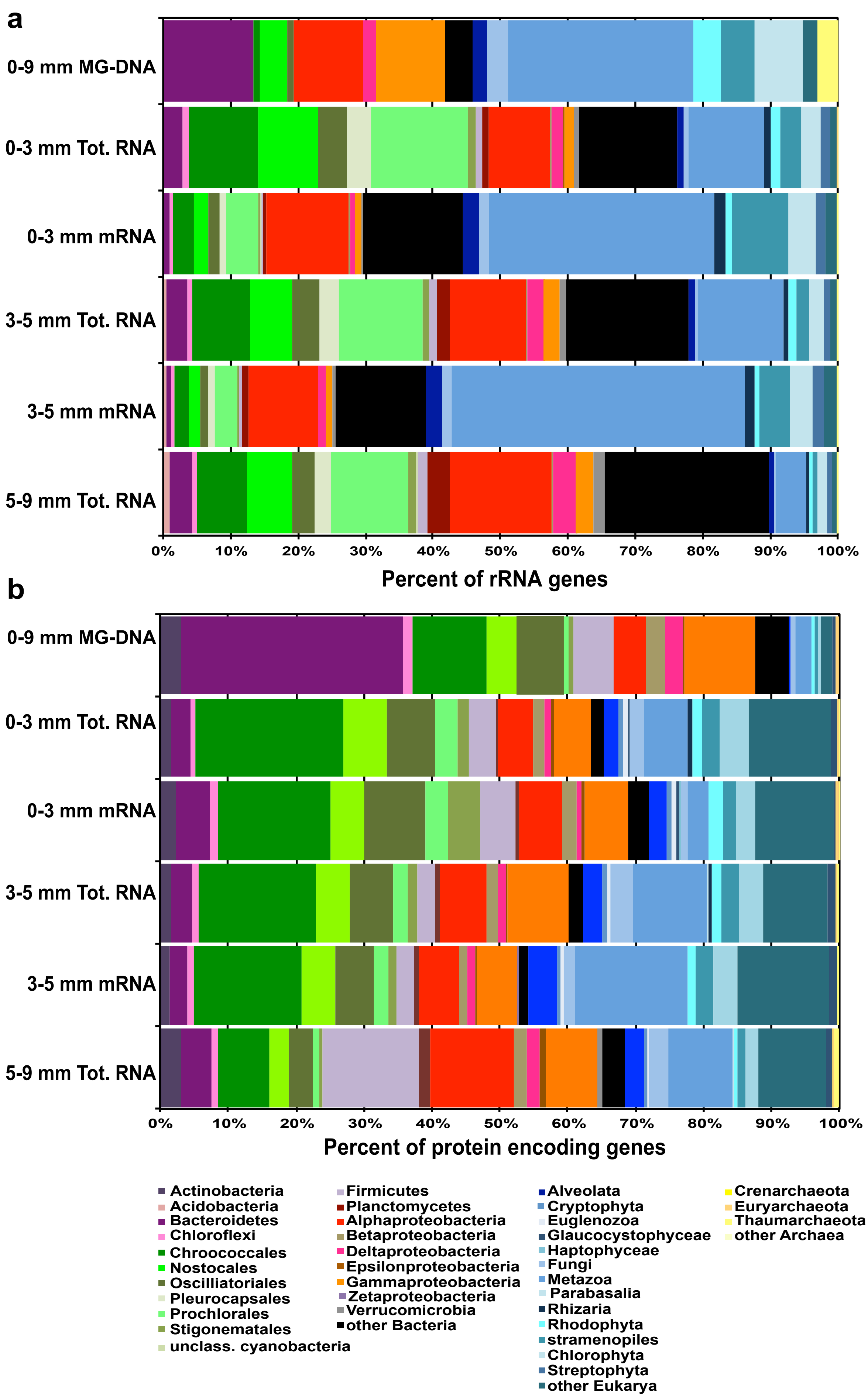

Supplemental Fig. S2

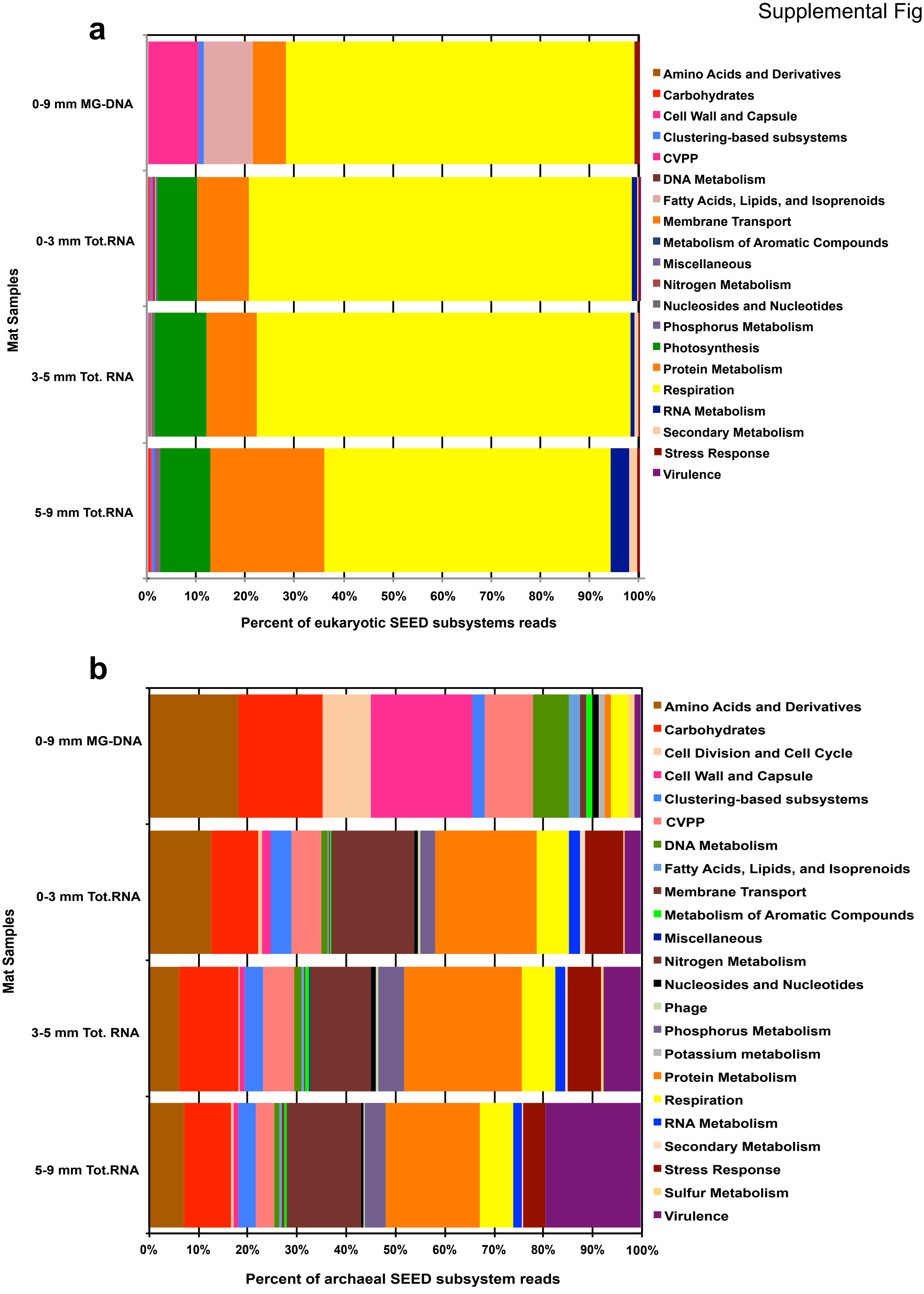

Supplemental Fig. S3

a

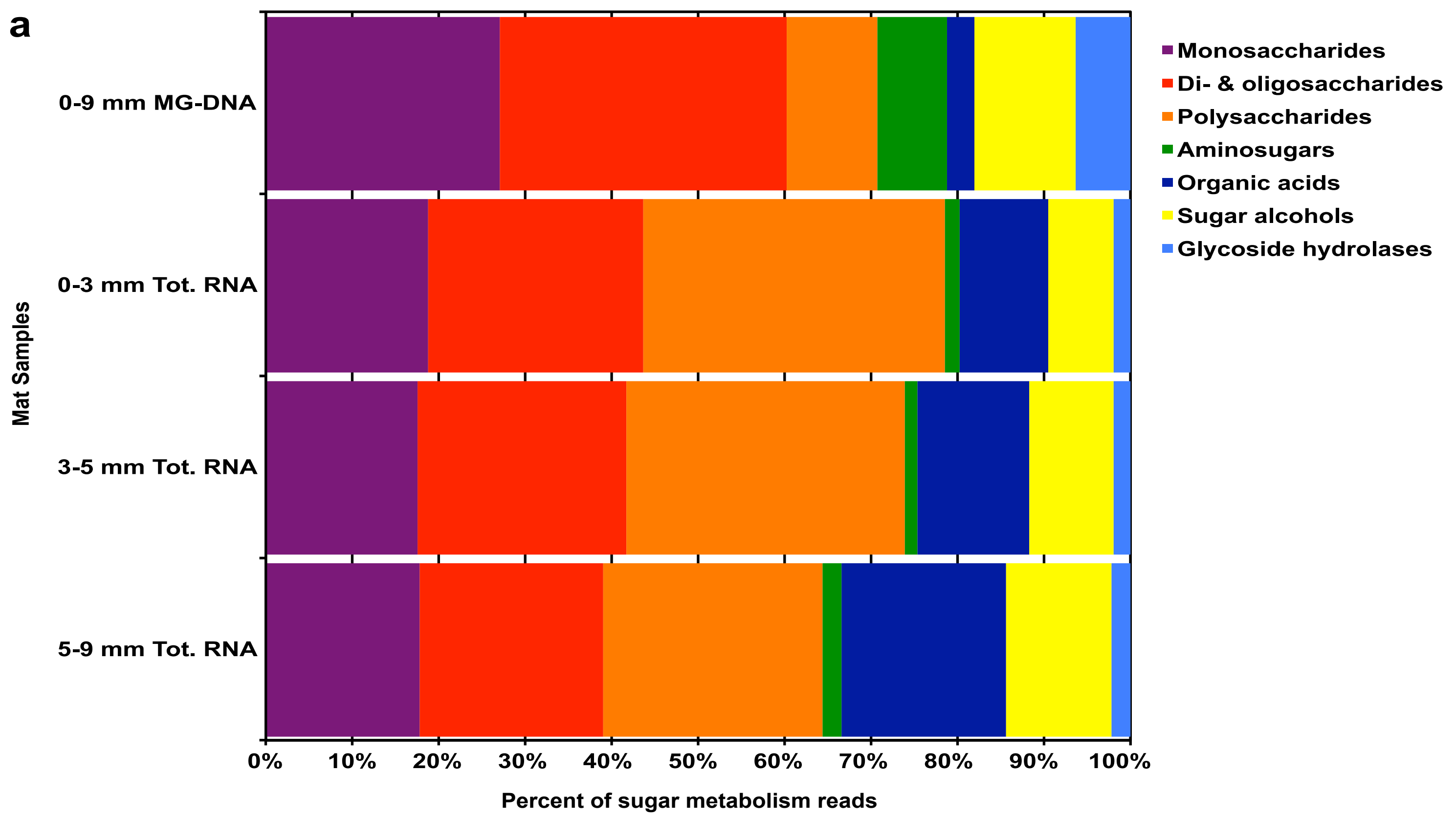

b

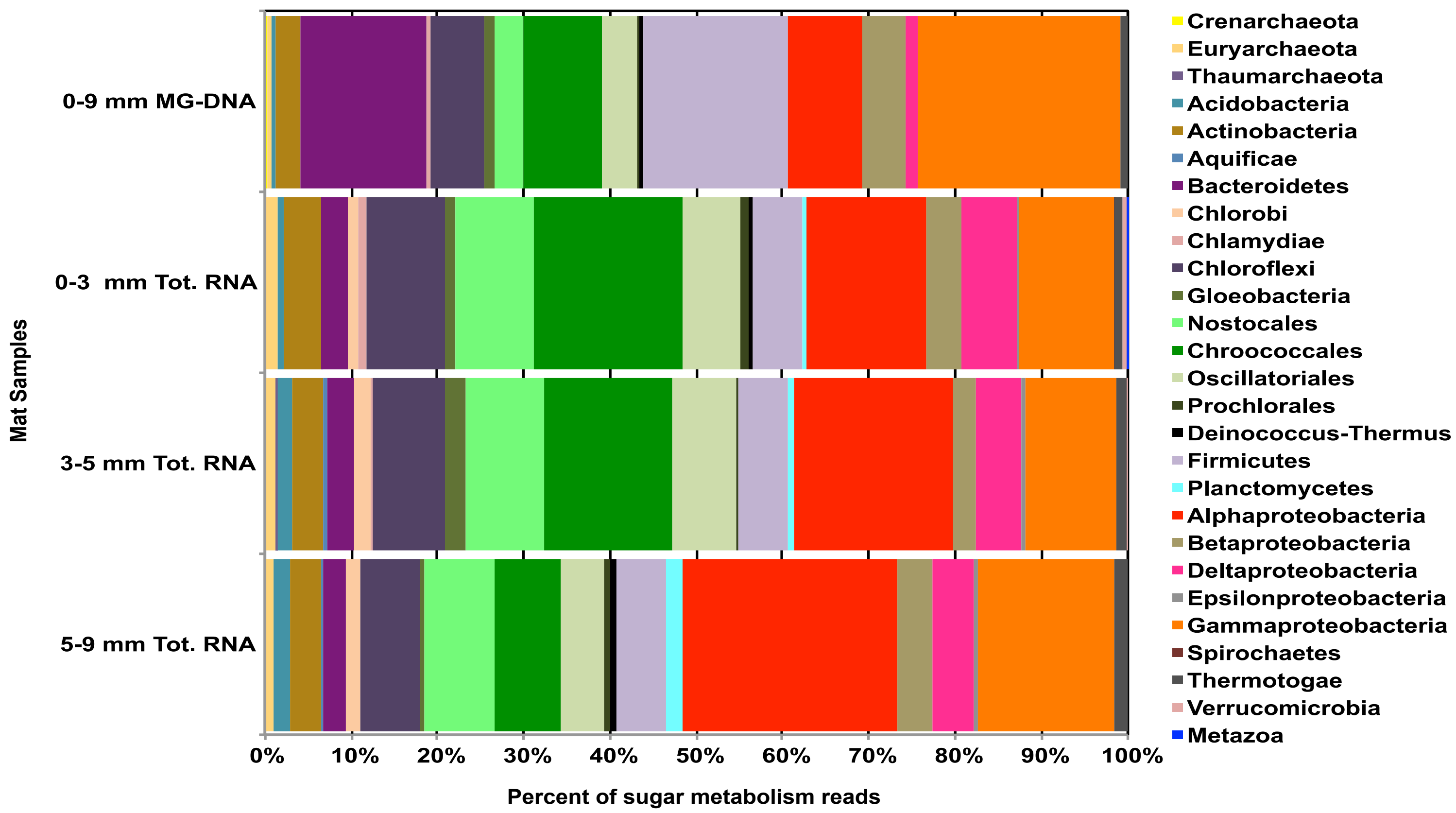

Supplementary Table S1. List of the KEGG proteins involved in specific energy transformation pathways targeted in this study.

KEGG pathway	KO	KEGG definition
photosynthesis	K02703	photosystem II P680 reaction center D1 protein
photosynthesis	K02704	photosystem II CP47 chlorophyll apoprotein
photosynthesis	K02705	photosystem II CP43 chlorophyll apoprotein
photosynthesis	K02706	photosystem II P680 reaction center D2 protein
photosynthesis	K02707	photosystem II cytochrome b559 subunit alpha
photosynthesis	K02708	photosystem II cytochrome b559 subunit beta
photosynthesis	K02709	photosystem II PsbH protein
photosynthesis	K02711	photosystem II PsbJ protein
photosynthesis	K02713	photosystem II PsbL protein
photosynthesis	K02716	photosystem II oxygen-evolving enhancer protein 1
photosynthesis	K02717	photosystem II oxygen-evolving enhancer protein 2
photosynthesis	K08901	photosystem II oxygen-evolving enhancer protein 3
photosynthesis	K03542	photosystem II 22kDa protein
photosynthesis	K02719	photosystem II PsbU protein
photosynthesis	K02720	photosystem II cytochrome c550
photosynthesis	K02723	photosystem II PsbY protein
photosynthesis	K02724	photosystem II PsbZ protein
photosynthesis	K08902	photosystem II Psb27 protein
photosynthesis	K08903	photosystem II 13kDa protein
photosynthesis	K02689	photosystem I P700 chlorophyll a apoprotein A1
photosynthesis	K02690	photosystem I P700 chlorophyll a apoprotein A2
photosynthesis	K02691	photosystem I subunit VII
photosynthesis	K02692	photosystem I subunit II
photosynthesis	K02693	photosystem I subunit IV
photosynthesis	K02694	photosystem I subunit III
photosynthesis	K02697	photosystem I subunit IX
photosynthesis	K02698	photosystem I subunit X
photosynthesis	K02699	photosystem I subunit XI
photosynthesis	K02288	phycocyanobilin lyase alpha subunit [EC:4.-.-.-]
photosynthesis	K02290	phycobilisome rod-core linker protein
photosynthesis	K05376	phycoerythrin alpha chain
photosynthesis	K05378	phycoerythrin-associated linker protein
photosynthesis	K05380	phycoerythrin-associated linker protein
photosynthesis	K05384	bilin biosynthesis protein
RuBisCo	K01601	ribulose-bisphosphate carboxylase large chain [EC:4.1.1.39]
RuBisCo	K01602	ribulose-bisphosphate carboxylase small chain [EC:4.1.1.39]
Nitrogen fixation	K02586	nitrogenase molybdenum-iron protein alpha chain NifD [EC:1.18.6.1]
Nitrogen fixation	K02591	nitrogenase molybdenum-iron protein beta chain NifK [EC:1.18.6.1]
Nitrogen fixation	K02588	nitrogenase iron protein NifH [EC:1.18.6.1]
Oxidative phosphorylation	K00330	NADH-quinone oxidoreductase subunit A [EC:1.6.5.3]
Oxidative phosphorylation	K00331	NADH-quinone oxidoreductase subunit B [EC:1.6.5.3]

Oxidative phosphorylation	K00332	NADH-quinone oxidoreductase subunit C [EC:1.6.5.3]
Oxidative phosphorylation	K00333	NADH-quinone oxidoreductase subunit D [EC:1.6.5.3]
Oxidative phosphorylation	K13378	NADH-quinone oxidoreductase subunit C/D [EC:1.6.5.3]
Oxidative phosphorylation	K13380	NADH-quinone oxidoreductase subunit B/C/D [EC:1.6.5.3]
Oxidative phosphorylation	K00334	NADH-quinone oxidoreductase subunit E [EC:1.6.5.3]
Oxidative phosphorylation	K00335	NADH-quinone oxidoreductase subunit F [EC:1.6.5.3]
Oxidative phosphorylation	K00336	NADH-quinone oxidoreductase subunit G [EC:1.6.5.3]
Oxidative phosphorylation	K00337	NADH-quinone oxidoreductase subunit H [EC:1.6.5.3]
Oxidative phosphorylation	K00338	NADH-quinone oxidoreductase subunit I [EC:1.6.5.3]
Oxidative phosphorylation	K00339	NADH-quinone oxidoreductase subunit J [EC:1.6.5.3]
Oxidative phosphorylation	K00340	NADH-quinone oxidoreductase subunit K [EC:1.6.5.3]
Oxidative phosphorylation	K00341	NADH-quinone oxidoreductase subunit L [EC:1.6.5.3]
Oxidative phosphorylation	K00342	NADH-quinone oxidoreductase subunit M [EC:1.6.5.3]
Oxidative phosphorylation	K00343	NADH-quinone oxidoreductase subunit N [EC:1.6.5.3]
Oxidative phosphorylation	K05572	NAD(P)H-quinone oxidoreductase subunit 1 [EC:1.6.5.3]
Oxidative phosphorylation	K05573	NAD(P)H-quinone oxidoreductase subunit 2 [EC:1.6.5.3]
Oxidative phosphorylation	K05574	NAD(P)H-quinone oxidoreductase subunit 3 [EC:1.6.5.3]
Oxidative phosphorylation	K05575	NAD(P)H-quinone oxidoreductase subunit 4 [EC:1.6.5.3]
Oxidative phosphorylation	K05576	NAD(P)H-quinone oxidoreductase subunit 4L [EC:1.6.5.3]
Oxidative phosphorylation	K05577	NAD(P)H-quinone oxidoreductase subunit 5 [EC:1.6.5.3]
Oxidative phosphorylation	K05578	NAD(P)H-quinone oxidoreductase subunit 6 [EC:1.6.5.3]
Oxidative phosphorylation	K05579	NAD(P)H-quinone oxidoreductase subunit H [EC:1.6.5.3]
Oxidative phosphorylation	K05580	NAD(P)H-quinone oxidoreductase subunit I [EC:1.6.5.3]
Oxidative phosphorylation	K05581	NAD(P)H-quinone oxidoreductase subunit J [EC:1.6.5.3]
Oxidative phosphorylation	K05582	NAD(P)H-quinone oxidoreductase subunit K [EC:1.6.5.3]
Oxidative phosphorylation	K05584	NAD(P)H-quinone oxidoreductase subunit M [EC:1.6.5.3]
Oxidative phosphorylation	K05585	NAD(P)H-quinone oxidoreductase subunit N [EC:1.6.5.3]
Oxidative phosphorylation	K05586	bidirectional [NiFe] hydrogenase diaphorase subunit [EC:1.6.5.3]
Oxidative phosphorylation	K05587	bidirectional [NiFe] hydrogenase diaphorase subunit [EC:1.6.5.3]
Oxidative phosphorylation	K05588	bidirectional [NiFe] hydrogenase diaphorase subunit [EC:1.6.5.3]
Oxidative phosphorylation	K03883	NADH-ubiquinone oxidoreductase chain 5 [EC:1.6.5.3]
Oxidative phosphorylation	K03934	NADH dehydrogenase (ubiquinone) Fe-S protein 1 [EC:1.6.5.3 1.6.99.3]
Oxidative phosphorylation	K03935	NADH dehydrogenase (ubiquinone) Fe-S protein 2 [EC:1.6.5.3 1.6.99.3]
Oxidative phosphorylation	K03936	NADH dehydrogenase (ubiquinone) Fe-S protein 3 [EC:1.6.5.3 1.6.99.3]
Oxidative phosphorylation	K03937	NADH dehydrogenase (ubiquinone) Fe-S protein 4 [EC:1.6.5.3 1.6.99.3]
Oxidative phosphorylation	K03939	NADH dehydrogenase (ubiquinone) Fe-S protein 6 [EC:1.6.5.3 1.6.99.3]
Oxidative phosphorylation	K03940	NADH dehydrogenase (ubiquinone) flavoprotein 1 [EC:1.6.5.3 1.6.99.3]
Oxidative phosphorylation	K03941	NADH dehydrogenase (ubiquinone) Fe-S protein 8 [EC:1.6.5.3 1.6.99.3]
Oxidative phosphorylation	K03942	NADH dehydrogenase (ubiquinone) flavoprotein 1 [EC:1.6.5.3 1.6.99.3]
Oxidative phosphorylation	K03943	NADH dehydrogenase (ubiquinone) flavoprotein 2 [EC:1.6.5.3 1.6.99.3]
Oxidative phosphorylation	K03948	NADH dehydrogenase (ubiquinone) 1 alpha subcomplex 4 [EC:1.6.5.3 1.6.99.3]
Oxidative phosphorylation	K03950	NADH dehydrogenase (ubiquinone) 1 alpha subcomplex 6 [EC:1.6.5.3 1.6.99.3]
Oxidative phosphorylation	K03952	NADH dehydrogenase (ubiquinone) 1 alpha subcomplex 8 [EC:1.6.5.3 1.6.99.3]
Oxidative phosphorylation	K03953	NADH dehydrogenase (ubiquinone) 1 alpha subcomplex 9 [EC:1.6.5.3 1.6.99.3]
Oxidative phosphorylation	K03955	NADH dehydrogenase (ubiquinone) 1 alpha/beta subcomplex 1 [EC:1.6.5.3 1.6.99.3]

Oxidative phosphorylation	K11352	NADH dehydrogenase (ubiquinone) 1 alpha subcomplex 12 [EC:1.6.5.3 1.6.99.3]
Oxidative phosphorylation	K03960	NADH dehydrogenase (ubiquinone) 1 beta subcomplex 4 [EC:1.6.5.3 1.6.99.3]
Oxidative phosphorylation	K03965	NADH dehydrogenase (ubiquinone) 1 beta subcomplex 9 [EC:1.6.5.3 1.6.99.3]
Oxidative phosphorylation	K03966	NADH dehydrogenase (ubiquinone) 1 beta subcomplex 10 [EC:1.6.5.3 1.6.99.3]
Oxidative phosphorylation	K00356	NADH dehydrogenase [EC:1.6.99.3]
Oxidative phosphorylation	K03885	NADH dehydrogenase [EC:1.6.99.3]
Oxidative phosphorylation	K05903	NADH dehydrogenase (quinone) [EC:1.6.99.5]
Oxidative phosphorylation	K00239	succinate dehydrogenase flavoprotein subunit [EC:1.3.99.1]
Oxidative phosphorylation	K00240	succinate dehydrogenase iron-sulfur subunit [EC:1.3.99.1]
Oxidative phosphorylation	K00241	succinate dehydrogenase cytochrome b556 subunit
Oxidative phosphorylation	K00242	succinate dehydrogenase membrane anchor subunit
Oxidative phosphorylation	K00244	fumarate reductase flavoprotein subunit [EC:1.3.99.1]
Oxidative phosphorylation	K00245	fumarate reductase iron-sulfur subunit [EC:1.3.99.1]
Oxidative phosphorylation	K00236	succinate dehydrogenase (ubiquinone) cytochrome b560 subunit
Oxidative phosphorylation	K00234	succinate dehydrogenase (ubiquinone) flavoprotein subunit [EC:1.3.5.1]
Oxidative phosphorylation	K00235	succinate dehydrogenase (ubiquinone) iron-sulfur subunit [EC:1.3.5.1]
Oxidative phosphorylation	K00411	ubiquinol-cytochrome c reductase iron-sulfur subunit [EC:1.10.2.2]
Oxidative phosphorylation	K00412	ubiquinol-cytochrome c reductase cytochrome b subunit
Oxidative phosphorylation	K00413	ubiquinol-cytochrome c reductase cytochrome c1 subunit
Oxidative phosphorylation	K00416	ubiquinol-cytochrome c reductase subunit 6
Oxidative phosphorylation	K00418	ubiquinol-cytochrome c reductase subunit 8
Oxidative phosphorylation	K02301	protoheme IX farnesyltransferase [EC:2.5.1.-]
Oxidative phosphorylation	K02257	protoheme IX farnesyltransferase [EC:2.5.1.-]
Oxidative phosphorylation	K02276	cytochrome c oxidase subunit III [EC:1.9.3.1]
Oxidative phosphorylation	K02274	cytochrome c oxidase subunit I [EC:1.9.3.1]
Oxidative phosphorylation	K02275	cytochrome c oxidase subunit II [EC:1.9.3.1]
Oxidative phosphorylation	K02262	cytochrome c oxidase subunit 3
Oxidative phosphorylation	K02256	cytochrome c oxidase subunit 1 [EC:1.9.3.1]
Oxidative phosphorylation	K02261	cytochrome c oxidase subunit 2
Oxidative phosphorylation	K02263	cytochrome c oxidase subunit 4
Oxidative phosphorylation	K02264	cytochrome c oxidase subunit 5a
Oxidative phosphorylation	K02265	cytochrome c oxidase subunit 5b
Oxidative phosphorylation	K02267	cytochrome c oxidase subunit 6b
Oxidative phosphorylation	K02258	cytochrome c oxidase assembly protein subunit 11
Oxidative phosphorylation	K02259	cytochrome c oxidase assembly protein subunit 15
Oxidative phosphorylation	K00404	cytochrome c oxidase cbb3-type subunit I [EC:1.9.3.1]
Oxidative phosphorylation	K00405	cytochrome c oxidase cbb3-type subunit II
Oxidative phosphorylation	K00406	cytochrome c oxidase cbb3-type subunit III
Oxidative phosphorylation	K02826	cytochrome aa3-600 menaquinol oxidase subunit II [EC:1.10.3.12]
Oxidative phosphorylation	K02299	cytochrome o ubiquinol oxidase subunit III [EC:1.10.3.-]
Oxidative phosphorylation	K02298	cytochrome o ubiquinol oxidase subunit I [EC:1.10.3.-]
Oxidative phosphorylation	K02297	cytochrome o ubiquinol oxidase subunit II [EC:1.10.3.-]
Oxidative phosphorylation	K00425	cytochrome d ubiquinol oxidase subunit I [EC:1.10.3.-]
Oxidative phosphorylation	K00426	cytochrome d ubiquinol oxidase subunit II [EC:1.10.3.-]
Oxidative phosphorylation	K02114	F-type H+-transporting ATPase subunit epsilon [EC:3.6.3.14]

Oxidative phosphorylation	K02112	F-type H+-transporting ATPase subunit beta [EC:3.6.3.14]
Oxidative phosphorylation	K02115	F-type H+-transporting ATPase subunit gamma [EC:3.6.3.14]
Oxidative phosphorylation	K02111	F-type H+-transporting ATPase subunit alpha [EC:3.6.3.14]
Oxidative phosphorylation	K02113	F-type H+-transporting ATPase subunit delta [EC:3.6.3.14]
Oxidative phosphorylation	K02109	F-type H+-transporting ATPase subunit b [EC:3.6.3.14]
Oxidative phosphorylation	K02110	F-type H+-transporting ATPase subunit c [EC:3.6.3.14]
Oxidative phosphorylation	K02108	F-type H+-transporting ATPase subunit a [EC:3.6.3.14]
Oxidative phosphorylation	K02134	F-type H+-transporting ATPase subunit delta [EC:3.6.3.14]
Oxidative phosphorylation	K02133	F-type H+-transporting ATPase subunit beta [EC:3.6.3.14]
Oxidative phosphorylation	K02136	F-type H+-transporting ATPase subunit gamma [EC:3.6.3.14]
Oxidative phosphorylation	K02132	F-type H+-transporting ATPase subunit alpha [EC:3.6.3.14]
Oxidative phosphorylation	K02128	F-type H+-transporting ATPase subunit c [EC:3.6.3.14]
Oxidative phosphorylation	K02127	F-type H+-transporting ATPase subunit b [EC:3.6.3.14]
Oxidative phosphorylation	K02117	V-type H+-transporting ATPase subunit A [EC:3.6.3.14]
Oxidative phosphorylation	K02118	V-type H+-transporting ATPase subunit B [EC:3.6.3.14]
Oxidative phosphorylation	K02121	V-type H+-transporting ATPase subunit E [EC:3.6.3.14]
Oxidative phosphorylation	K02146	V-type H+-transporting ATPase subunit AC39 [EC:3.6.3.14]
Oxidative phosphorylation	K02151	V-type H+-transporting ATPase subunit F [EC:3.6.3.14]
Oxidative phosphorylation	K02148	V-type H+-transporting ATPase subunit C [EC:3.6.3.14]
Oxidative phosphorylation	K02145	V-type H+-transporting ATPase subunit A [EC:3.6.3.14]
Oxidative phosphorylation	K02147	V-type H+-transporting ATPase subunit B [EC:3.6.3.14]
Oxidative phosphorylation	K02155	V-type H+-transporting ATPase 16kDa proteolipid subunit [EC:3.6.3.14]
Oxidative phosphorylation	K03661	V-type H+-transporting ATPase 21kDa proteolipid subunit [EC:3.6.3.14]
Oxidative phosphorylation	K02154	V-type H+-transporting ATPase subunit I [EC:3.6.3.14]
Oxidative phosphorylation	K02149	V-type H+-transporting ATPase subunit D [EC:3.6.3.14]
Oxidative phosphorylation	K02152	V-type H+-transporting ATPase subunit G [EC:3.6.3.14]
Oxidative phosphorylation	K02144	V-type H+-transporting ATPase 54 kD subunit [EC:3.6.3.14]
Oxidative phosphorylation	K01544	non-gastric H+/K+-exchanging ATPase [EC:3.6.3.10]
Oxidative phosphorylation	K01507	inorganic pyrophosphatase [EC:3.6.1.1]
Oxidative phosphorylation	K11726	nucleosome-remodeling factor 38 kDa subunit [EC:3.6.1.1]
Oxidative phosphorylation	K00937	polyphosphate kinase [EC:2.7.4.1]
